# Influence of COVID-19 on Health-Related Quality of Life and the Perception of Being Vaccinated to Prevent COVID-19: An Approach for Community Pharmacists from Romania and Bulgaria

**DOI:** 10.3390/jcm10040864

**Published:** 2021-02-19

**Authors:** Adina Turcu-Stiolica, Maria Bogdan, Mihaela-Simona Subtirelu, Andreea-Daniela Meca, Adriana-Elena Taerel, Irina Iaru, Maria Kamusheva, Guenka Petrova

**Affiliations:** 1 Department of Pharmacoeconomics, University of Medicine and Pharmacy of Craiova, 200349 Craiova, Romania; adina.turcu@gmail.com (A.T.-S.); mihaela.subtirelu@yahoo.com (M.-S.S.); 2 Department of Pharmacology, University of Medicine and Pharmacy of Craiova, 200349 Craiova, Romania; andreea_mdc@yahoo.com; 3 Department of Pharmaceutical Management, University of Medicine and Pharmacy “Carol Davila”, 020021 Bucharest, Romania; adriana.taerel@yahoo.com; 4 Department of Pharmacology, Physiology and Pathophysiology, Iuliu Hatieganu University of Medicine and Pharmacy, 400012 Cluj-Napoca, Romania; cazacuirina@yahoo.com; 5 Department of Organization and Economics of Pharmacy, Medical University-Sofia, 1000 Sofia, Bulgaria; gpetrova@pharmfac.mu-sofia.bg

**Keywords:** quality of life, 15D, COVID-19, vaccination, community pharmacists

## Abstract

Community pharmacists are essential front-line health workers, involved in relieving the COVID-19 burden. Their health-related quality of life status needs to be assessed, as lower levels could affect their functioning. In order to evaluate the current status of community pharmacists’ quality of life from Romania and Bulgaria during the COVID-19 pandemic, and to identify factors associated with their decision on being vaccinated to prevent COVID-19, an online survey involving 395 community pharmacists was conducted from 15th July 2020 to 15th August 2020. The 15D instrument was used for quality-of-life assessment. The pharmacists’ recommendations for vitamin C and D intake during the COVID-19 pandemic were also analyzed in order to promote future training programs for community pharmacists. Descriptive statistics, comparative analyses between pharmacists from Romania and Bulgaria, and multiple correlation analyses were performed on the collected data. Significant differences were observed for the level of quality of life between the two groups of pharmacists according to their age; smaller values, directly correlated with their age (total 15D score and age: Spearman r = 0.168, *p* = 0.022), were obtained for Bulgarian pharmacists regarding sleeping, usual activities, mental function, discomfort and symptoms, depression, distress. The perception of being vaccinated did not differ between Romanian and Bulgarian pharmacists, as almost 50% agreed to vaccination (*p* = 0.7542). Their willingness to vaccinate was correlated with vitamin D usage (*p* = 0.0134), rather than with vitamin C (*p* = 0.4157). No other significant associations were found between willingness to get vaccinated to prevent COVID-19 and other characteristics (age, gender, income, quality-of-life markers). Evidence-based interventions are required to enhance the health-related quality of life of community pharmacists involved in the first line of the COVID-19 pandemic.

## 1. Introduction

Over a year has passed since the first declared case of COVID-19, a life-threatening infection, on 31st of December 2019, and all of our socio-economic life aspects have been affected while facing this worldwide pandemic [1,2,3,4]. Frontline health workers have reached unprecedented anxiety levels, as information regarding contagiousness and pharmacotherapeutic management remains uncertain [1]. Long-lasting immense stress and panic, as well as physical exhaustion, could lead to post-traumatic disorders or depression, with a higher prevalence among healthcare workers [1]. Moreover, the performance of health care professionals could also be affected due to increased workload and fear of self-infection, social rejection, or even transmission to family members [5,6].

**Figure 1:**
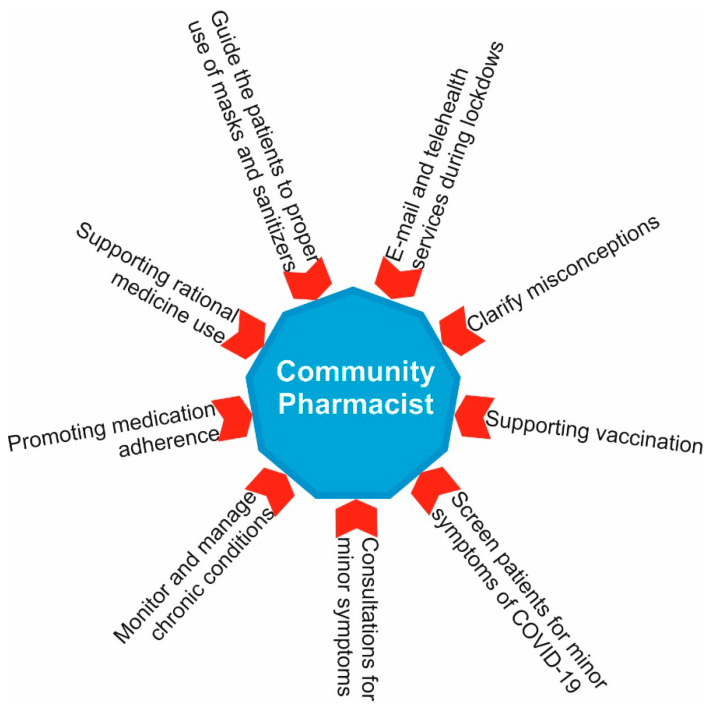
Community pharmacy services during COVID-19 pandemic.

Inter-professional collaboration among healthcare settings, including community pharmacies, is highly recommended in order to face and manage the COVID-19 pandemic [2]. During the Ebola epidemics, for example, effective communication between physicians and community pharmacists was noted as a good control measure in avoiding the infection spread [7]. Pharmacists are exposed daily through face-to-face interactions with individuals [3], but are often neglected as frontline health care providers, although they represent accessible, essential health care workers [6,7,8,9]. Although pharmaceutical services cannot be offered from a remote location, little information about COVID-19 exposure and prevalence among community pharmacists is noted [9,10], which could be underestimated [7,10]. However, a higher risk of exposure to COVID-19-infected individuals involves a greater psychological impact [11]. Permanent fear of infection due to insufficient social distancing in pharmacies, aggressive patient behavior and higher stress levels could affect cognitive functioning in pharmacists and could weaken their attention [1,12]. Community pharmacists have also been involved in the screening and triage of COVID-19 patients (Figure 1), as well as in ensuring continuity of care for non-COVID-19 illnesses and promoting pharmacological adherence, which further increased the negative impact on their well-being [2,9]. However, pharmacists have shown a trustworthy attitude regarding the adoption of protective measures (Figure 1), despite the increased risk of burnout [8,10,12]. They even provided surveillance of suspicious cases, symptoms-related counselling and advice to patients on whether they should be quarantined or not, depending on their clinical manifestations (Figure 1) [7]. Community pharmacists also improved medical education among their patients by using telehealth consulting and new technologies [2]. Moreover, pharmacists maintained contact, through different and accessible smart mobile applications, e-mails, video calls and written text messages, with all individuals who requested pharmaceutical assistance (Figure 1) [2]. Continuous pharmaceutical monitoring and communication with patients not only supported rational medicine use, but also ensured constant clarification of various misconceptions regarding prophylactic or long-term curative treatments (Figure 1) [2]. Thereby, community pharmacists helped reduce unnecessary visits to health departments, which were already overwhelmed, ensuring a cost-effective approach to the COVID-19 pandemic [7]. A systematic review conducted in July 2019 underlined the importance and involvement of pharmacists in public threat recovery [6]; therefore, their proper mental health and quality of life are highly needed.

Community pharmacists are involved in pharmacovigilance activities and are able to educate and counsel individuals regarding both the pharmacotherapeutic approach of the COVID-19 infection and the proper use of personal protective equipment and sanitizers (Figure 1) [2,7,12]. Pharmacists are also able to communicate effectively in order to combat the flood of misinformation regarding COVID-19 and discourage dangerous self-medication [7,8,11,12,13]. Moreover, pharmacists could play a key role in the prevention and containment of COVID-19 pandemic, promoting patients’ adherence to prophylactic immunomodulatory treatment (such as vitamins C and D); they could also promote vaccination and support vaccinovigilance (Figure 1) [2,7,8,13].

The European Medicines Agency (EMA) authorized Cominarty (developed by BioNTech and Pfizer) as the first vaccine used for prevention of COVID-19, in people aged over 16 years, in European countries, on 21st December 2020 [14]. Another conditional marketing authorization was given on 6th January 2021 for COVID-10 vaccine Moderna, recommended in individuals from 18 years of age [14]. The first two approved vaccines are based on the mRNA technique, preparing the human body to generate a proper immune response in case of infection. The most recent recommended vaccine for European authorization was the one from AstraZeneca, on 29th January 2021, based on modified adenovirus. All three vaccines will be monitored in both European countries (Romania [15] and Bulgaria [16]) through pharmacovigilance online systems, and intensively supported and promoted by pharmacists.

Vitamin C (ascorbic acid) cannot be synthesized by the human organism, but it is found in many fruits and vegetables [17,18,19] and is used as a supplement, alone or in combination with other vitamins and minerals [20]. Vitamin D exists in two forms: vitamin D2 (found in plants) and vitamin D3 (found in food and synthesized in the skin under sunlight ultraviolet-B ray exposure), both of which are available in dietary supplements [21,22]. An indoor lifestyle, sun avoidance for health or cultural reasons, and a modern diet based on highly processed food are significant contributors to the evolution of global vitamin D deficiency [23]. Even more, due to the long period of lockdown and following limitation of activities outside the home, vitamin D intake is beneficial during the pandemic. Both vitamin C and vitamin D supplementation has demonstrated positive effects on the immune system and on respiratory tract infections [24,25].

Pharmacists represent the first point of contact with individuals who need psychiatric assistance [12]. Robinson et al. emphasizes that in order to assist patients with behavioural health conditions (such as concern, fear, insomnia, fatigue, anger, frustration, intense anxiety, panic attacks, depression) and to help relieve the pandemic pressure, community pharmacists should be trained to first manage their own psychological disorders [3,4,5,6,9,12]. Nevertheless, self-care (physical activity, proper eating and sleep hygiene, mindfulness techniques) and resilience need greater promotion among pharmacists [11,12]. Multidisciplinary mental health services, such as training programs and careful evaluation of the pharmacy staff, should be initiated and provided for pharmacists as soon as possible [9,12].

Romania declared a COVID-19 lockdown from 16th March 2020 to 15th May 2020, whereas in Bulgaria the state of emergency was between 13th March 2020 and 13th May 2020. In both countries, it was followed by an alert period with many limitations and restrictions, which still continues. The reason we choose these two countries for the study was because both are from the Central and Eastern European region and have similar drug policies and health legislation [26].

Our aim was to analyze the health-related quality of life (HRQoL) of community pharmacists from Romania and Bulgaria during the COVID-19 pandemic and to identify factors associated with perception of being vaccinated against COVID-19, in order to provide evidence-based interventions to community pharmacists to improve vaccine use. Associations between HRQoL, demographics, pharmacists’ recommendations for vitamin C and D intake during the pandemic, and perception of being vaccinated against COVID-19 were evaluated. At the same time, based on multiple clinical trials about the vitamin C/vitamin D relation with COVID- 19, we aimed to assess the insight of the community pharmacists regarding the recommendation of over-the-counter drugs with vitamin C and D during the COVID-19 pandemic.

## 2. Materials and Methods

### 2.1. Study Design

The cross-sectional study was conducted from 15th July 2020 to 15th August 2020. The online survey was developed using Google Forms and included three main sections: socio-demographic characteristics, 15D instrument and perception towards COVID-19 vaccination (Supplementary file_Questionnaire). The online survey was anonymous and confidential. As dissemination channels, we used pharmacist groups from social media, such as Facebook, and emails to our former students.

The socio-demographic section was composed of items exploring age, gender, marital status, experience as a pharmacist, income working as a pharmacist, and pharmacy specialization. Monthly income working as a pharmacist was stratified into low (less than 600 Euro), medium (600–1000 Euro), or high (more than 1000 Euro).

The second section of the questionnaire assessed the HRQoL using the 15D instrument [27,28]. The 15D questionnaire comprises 15 dimensions (mobility, vision, hearing, breathing, sleeping, eating, speech, excretion, usual activities, mental function, discomfort and symptoms, depression, distress, vitality, and sexual activity), with each dimension being divided into five levels that range from no problems to severe difficulties. The single index score (15D score), representing the overall HRQoL on a 0–1 scale (1 = full health, 0 = being dead) and the dimension level values, reflecting the goodness of the levels relative to no problems on the dimension (=1) and to being dead (=0), are calculated from the questionnaire by using a set of population-based preference or utility weights.

The third section included five items specifically developed for the purpose of the study, which were related to vitamin C or D supplementation (self or recommended to others) during the COVID-19 pandemic and to vaccination against COVID-19.

### 2.2. Participants

The survey was addressed to pharmacists working in community pharmacies from Romania and Bulgaria.

To calculate an appropriate representative sample from the targeted population, convenience sampling was used [29]. Power analysis was conducted using G*Power 3 (alpha equal to 0.05) and a sample size of 176 pharmacists was calculated to demonstrate statistically significant results for the study [30]. Ethical approval for this study was obtained from the Ethics Committee of the University of Medicine and Pharmacy of Craiova (Registration no. 54/08.07.2020) according to the Declaration of Helsinki. All pharmacists provided electronic informed consent, starting with the first question of the survey.

### 2.3. Data Analysis

Continuous variables were summarized as mean ± standard deviation (SD) or median with interquartile range (IQR). Statistical differences were analyzed by Student’s *t-*test or Mann–Whitney test. Categorical variables were summarized as frequencies and percentages. Chi-squared test was performed to compare categorical variables as appropriate. We further assessed the association of continuous variables with 15D scores by Spearman’s correlation test. ANCOVA was used to capture association between continuous and categorical variables. We did not have missing data. Data were analyzed using GraphPad Prism 9.0 software (GraphPad Software, LLC, San Diego, CA, USA). The significance level was 0.05.

## 3. Results

The sample included 395 pharmacists working in community pharmacies with possible contacts with COVID-19 patients: 241 from Romania and 154 from Bulgaria. The results are shown in Table 1.

The characteristics of the two groups of pharmacists showed that the Romanian pharmacists were older, with a higher percentage of women, married people and specialists. When considering the income working as a pharmacist, there are statistically significant differences between the two groups; Bulgarian pharmacists have a higher income, even if they are less experienced.

Different pharmacy specialties exist in Romania and Bulgaria. Of the 108 specialist pharmacists from Romania, 47 (44%) were specialists in Clinical Pharmacy, 51 (47%) were specialists in General Pharmacy, and 10 (9%) were specialists in Pharmaceutical Laboratory. In Bulgaria, most pharmacists were specialists in Clinical Pharmacy (46%).

Table 2 shows the differences between the levels of quality of life of pharmacists from Romania and Bulgaria. We observed statistically significant differences regarding sleeping, usual activities, mental function, discomfort and symptoms, depression, distress and total 15D score, with low values for distress. The smaller values for Bulgarian pharmacists were directly correlated with age (for the association between total 15D score and age: Spearman r = 0.168, *p* = 0.022). A strong correlation was also observed between mental function and discomfort and symptoms (Spearman r = −0.81, *p* = 0.028).

We assessed whether gender had a significant effect on HRQoL outcomes and no associations were found: breathing (*p*-value = 0.060), sleeping (*p*-value = 0.302), speech (*p*-value = 0.191), excretion (*p*-value = 0.582), usual activities (*p*-value = 0.887), mental function (*p*-value = 0.065), discomfort and symptoms (*p*-value = 0.322), depression (*p*-value = 0.677), distress (*p*-value = 0.622), vitality (*p*-value = 0.809), sexual activities (*p*-value = 0.077) and HRQoL score (*p*-value = 0.661).

**Figure 2:**
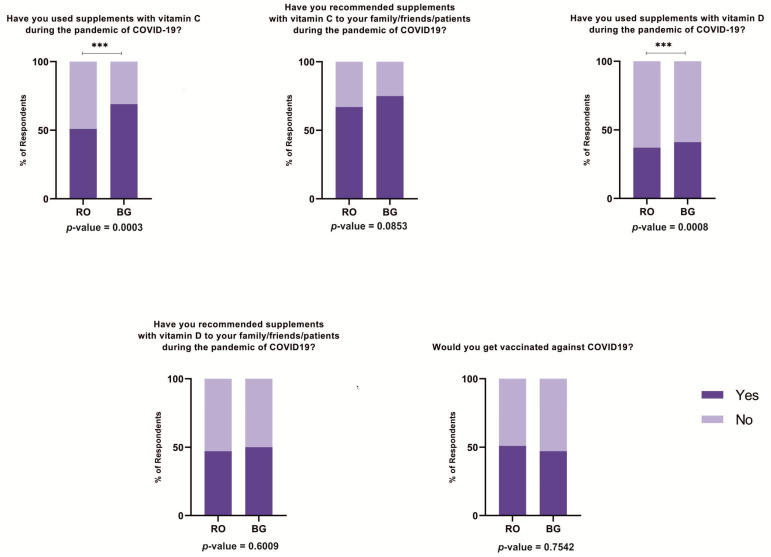
Perception towards COVID-19 vaccination and vitamin recommendation. *** *p*-value < 0.001.

To determine whether community pharmacists used and recommended supplements with vitamin C and D, their answers were assessed and presented in Figure 2. We observed statistical differences between the two groups in the case of using supplements with vitamin C and D: the pharmacists from Bulgaria used them more. The same trend was not maintained in terms of recommending these supplements to their family/friends/patients: the percentage of pharmacists that recommend was higher than the percentage of pharmacists that used these products, without differences between Romania and Bulgaria.

The community pharmacists from the two countries reported willingness to get vaccinated to prevent COVID-19 in almost the same percentage: 50% (*p =* 0.7542). A statistically significant association was found between the willingness to get vaccinated and the use of vitamin D during the COVID-19 pandemic (*p* = 0.0134). However, no association was found between the willingness to get vaccinated and the use of vitamin C during the COVID-19 pandemic (*p* = 0.4157). No other significant associations were found between the willingness to get vaccinated to prevent COVID-19 and other characteristics (age, gender, income, quality-of-life markers).

## 4. Discussion

The extreme psychological pressure felt by health care providers in the last year could affect not only patients, but also entire public healthcare systems [11,12]. However, pharmacists were required during this challenging pandemic to enlarge their traditional pharmaceutical activities and to ensure a primary point of care and triage for many patients [31,32,33].

A strong statistical correlation was found between discomfort and specific symptoms and mental functions among the pharmacists who participated in our study. Our study did not underline the higher vulnerability to psychological impact during the COVID-19 pandemic among female pharmacists, as other studies [34]. Batra et al. reported higher levels of depression, distress and behavioral dysfunctionalities among female health workers who have prolonged contact with patients [5]. The mental health impact of COVID-19 pandemic has been noticed in more than 50% of pharmacists, and some studies even highlighted higher rates of burnout among pharmacists in comparison with nurses and physicians [35]. Another study, conducted by Lange et al. [36], showed that approximately 35% of community pharmacists reported mental health disturbances (anxiety, stress, insomnia, sense of losing control, fear, hopelessness [37]), with females being more affected [36]. These results, coming from the very first studies analyzing the psychological impact of COVID-19 pandemic among community pharmacists, in the French region of Normandy [37], are not consistent with our results because of the different period of the studies. The same results about the level of quality of life of Romanian young physicians during COVID-19 pandemic were obtained, with no association between gender and distress [38].

Furthermore, our study included more specialists and older pharmacists from Romania, which could be characterized by better ability to assist, emergency preparedness and probable stress coping mechanisms due to longer experience [35]. Thereby, we noticed that Romanian pharmacists reported a better quality of life, whereas Bulgarian pharmacists reported more sleeping disturbances, distress and depression. Our statistical correlations proved that Bulgarian pharmacists had more difficulties in coping with usual activities, also underlining the mental pandemic burden.

Dror et al. [39] mentioned that first-line medical staff have less hesitancy towards vaccination against COVID-19 and its efficacy, while medical workers who have less or no contact with infected patients are more skeptical [39]. Vaccine acceptance could be increased not only by recommendations and proper information transmitted to patients by community pharmacists, who play an essential educational role, but also by pharmacists considering themselves eligible for vaccine acquiescence [39,40]. Although conditional marketing authorization for COVID-19 preventive vaccines is used in case of benefits outweighing risks for patients [14], clarifications regarding importance of vaccination are still needed among general population. Pharmacists are important pawns in the immunization campaign in both European countries included in our study, relieving the COVID-19 burden [9,41,42]. Most individuals are still skeptical and have misconceptions about receiving vaccines, even though more than 2.5 million deaths are prevented annually worldwide through vaccination programs [42]. Community pharmacists are not only able to increase vaccination rates through direct recommendations, but also by receiving approval to administer vaccines to patients [42]. One of the most important predictive factors for vaccine acceptance is the self-perception of a high risk for COVID-19, which could be achieved through medical education [39,41]. On the other hand, females are less likely to accept vaccination [39], a result consistent with our statistic, due to the higher prevalence of female pharmacists from both countries in our study.

This study offers an important insight into the quality of life of community pharmacists during COVID-19 pandemic and their perception towards vaccination. A limitation could be the lower age category for the community pharmacists who answered to this survey, which could be due to the dissemination channels we used. More professional experience accumulated over the years could have influenced the pharmacists’ answers to this survey. Additionally, the timeline for the survey dissemination may have influenced the pharmacists’ opinion about COVID-19 impact and, therefore, their responses may differ over time.

Another limitation of the study could be the comparison between the pharmacists’ income in the two countries, since the World Bank Country classification by income sets Bulgaria and Romania in different groups (Bulgaria is upper middle income and Romania is high income) [42]. Moreover, even if the World Bank Country sets Romania in the high-income group, the actual income for community pharmacists is still very low.

Our study represents one of the first published studies that, to our knowledge, analyzes COVID-19’s impact on quality of life among pharmacists in Romania and Bulgaria. We found only one study that underlined the pressure that community pharmacists are subjected to during lockdown [36]. Our work also stands as a comparison between two neighboring countries regarding psychological disturbance in community pharmacists, as front-line health-care workers. Moreover, our study is a first attempt to evaluate the consumption and recommendation of vitamin C and D among pharmacists during the pandemic.

For the European countries, an inverse correlation was described between national estimates of vitamin D levels and COVID-19 incidence [43,44] and mortality [43,44,45]. There is evidence that vitamin D is correlated with diminished risk and severity of COVID-19 infection through different mechanisms (decreasing the production of inflammatory cytokine, decreasing the survival and replication of viruses, preserving endothelial integrity, and augmenting angiotensin-converting enzyme 2 concentrations) [46]. The prophylactic vitamin D administration in the COVID-19 management was underscored [47]. Various studies were revised and the recommended daily dose by consensus during the COVID-19 pandemic, 2000 IU for teenagers and adults, is 20 times lower than the amount which must be taken for many months to cause toxicity [48].

Vitamin C and D supplements are easily available on the pharmaceutical market in each country, have a low risk of adverse effects, and are inexpensive. Both vitamins are recommended with precise doses for prophylaxis and treatment as part of the COVID-19 management protocol [49]. However, the results of ongoing clinical trials are expected to elucidate various aspects of the vitamin C/vitamin D relationship with COVID-19 (52 [50] and 71 [51], respectively of clinical trials registered at present on ClinicalTrials.gov). The outcomes of these clinical trials could also contribute to future training programs for community pharmacists in order to recommend vitamin C and D supplementation during the pandemic to better manage the COVID-19 burden.

Both Bulgaria and Romania are countries where a large-scale peak in COVID-19 infection would lead to overcrowded health facilities and a shortage of medical specialists. Reliability of information and control of fear and disinformation are important issues during the spread of the disease. Community pharmacies are one of the few places that are kept open for public service even during strict safety measures. They have a unique, credible role with ease of accessibility [2].

In Romania, reducing the program or patients’ access to it, the installation of protective panels or air purifiers, and disinfection of surfaces and personal objects at regular intervals (less than one hour), were measures adopted in order to ease the pandemic burden for both pharmacists and patients [52]. A survey conducted by Padureanu et al. between April and May 2020, in Romania, underlined that 52% of pharmacists were satisfied with the provided protection measures [53]. However, the same survey mentioned that 57% of pharmacists were afraid of COVID-19 exposure and infection, consistent with our results. Community pharmacists from Romania were allowed to release over-the-counter antibiotics for a correct and complete treatment of dental abscess during lockdown or whenever dental health services were blocked [52]. Elbeddini et al. showed that the increased distress was also caused by verbal abuse and harassment from patients demanding COVID-19 protocol drugs even if they did not have a prescription or were not diagnosed [9].

During the pandemic, pharmacists in Bulgaria were also in charge of additional administrative issues regarding the reporting of new paperless prescription forms, so-called “S blanks”, for patients who were treated with medication prescribed with special protocols and paid by the National Health Insurance Fund (NHIF) [54]. The purpose of this was for chronically ill patients to avoid visiting their general practitioners and specialists for the issuing of a new protocol [54]. This led to an extra workload for pharmacies.

The pharmacy owners from both European countries were obliged to ensure protective gloves, safety goggles and masks for all pharmacists [52,54]. However, the Bulgarian Pharmaceutical Union (BPU) and other non-profit associations, such as the Bulgarian Medicines Verification Organization, provided some quantity of protective equipment for BPU members [54]. No governmental help was ensured, since pharmacists work in the private sector.

Further follow-up studies conducted in both countries would be informative for the society and their results could be compared with the period analyzed in the current study. The inclusion of further neighboring countries in such a comparison might bring added value and could present a broader picture of how the COVID-19 pandemic has affected community pharmacists’ quality of life.

## 5. Conclusions

Evaluation of the health-related quality of life of pharmacists and assessment of the psychological impact of the COVID-19 pandemic leads to better recognition of their work and implicitly better management of community public health. The present study offers an important perspective regarding the perception of community pharmacists from Romania and Bulgaria towards vaccination and vitamins C and D recommendation. Our results could also support further strategies enhancing resilience during the COVID-19 pandemic among essential front-line healthcare workers such as pharmacists, which are often neglected.

## Figures and Tables

**Table 1:** Baseline characteristics of pharmacists from Romania and Bulgaria.

	Romania (*n* = 241)	Bulgaria (*n* = 154)	*p*-Value
Gender			
Men	18 (7%)	33 (21%)	<0.0001 *
Women	223 (93%)	121 (79%)	
Age ^, years	30 (26–37)	26 (25–32)	<0.0001 *
Marital status			
Married	117 (49%)	49 (32%)	0.0010 *
Not married	113 (47%)	100 (65%)	
Divorced	9 (4%)	4 (3%)	
Widower	2 (1%)	1 (1%)	
Professional experience ^, years	7.36 ± 8.3	6.2 ± 7.8	0.0124 *
Income working as pharmacist			
Low (less than 600 Euro)	95 (39%)	29 (19%)	<0.0001 *
High (more than 1000 Euro)	18 (7%)	33 (21%)	
Specialist pharmacists			<0.0001 *
Yes	108 (45%)	24 (16%)	
No	133 (55%)	130 (84%)	

*, significantly different (*p* < 0.05); ^, did not pass normality test.

**Table 2:** Health-related quality of lige (HRQoL) results.

Mean ± SD	Romania (*n* = 241)	Bulgaria (*n* = 154)	*p*-value
Mobility	1	1	>0.999
Vision	1	1	>0.999
Hearing	1	1	>0.999
Breathing	0.903 ± 0.143	0.928 ± 0.132	0.0702
Sleeping	0.896 ± 0.161	0.848 ± 0.207	0.0494 *
Eating	1	1	>0.999
Speech	0.982 ± 0.084	0.975 ± 0.083	0.1778
Excretion	0.976 ± 0.094	0.961 ± 0.104	0.0786
Usual activities	0.975 ± 0.113	0.961 ± 0.114	0.041 *
Mental function	0.979 ± 0.087	0.943 ± 0.136	0.0007 *
Discomfort and symptoms	0.921 ± 0.141	0.979 ± 0.094	<0.0001 *
Depression	0.933 ± 0.130	0.853 ± 0.197	<0.0001 *
Distress	0.844 ± 0.187	0.765 ± 0.217	0.0002 *
Vitality	0.881 ± 0.139	0.844 ± 0.185	0.1305
Sexual activities	0.925 ± 0.155	0.930 ± 0.141	0.14
Total 15D score	0.956 ± 0.051	0.936 ± 0.063	0.0024 *

*, significantly different (*p* < 0.05); SD, Standard Deviation.

## Data Availability

Data supporting reported results can be asked from the corresponding author.

## Supplementary Materials

The following are available online at https://www.mdpi.com/2077-0383/10/4/864/s1, Supplementary file_Questionnaire.
