# Supplementary material for: Influence of COVID-19 on Health-Related Quality of Life and the Perception of Being Vaccinated to Prevent COVID-19: An Approach for Community Pharmacists from Romania and Bulgaria
Source: J Clin Med. 2021 Feb 19;10(4):864. doi: 10.3390/jcm10040864 (PMC7923195; doi:10.3390/jcm10040864)
Supplement: Supplementary file 1 [file jcm-10-00864-s001.pdf]

Dear Pharmacists,

Please read carefully the questions and answer with the option that best describes your health condition at the moment. The aim is to study how the COVID19 pandemic affects your quality of life.

Your participation in this survey is voluntary, in compliance with European ethical recommendations on the absolute confidentiality of personal data collected in the survey, as well as the anonymity and security of participants (Regulation (EU) 2016/679 on the protection of individuals with regard to the processing of personal data).

If you have any questions regarding the study, your personal rights, you can reach by e-mail the investigators: adina.turcu@gmail.com or mkamusheva@pharmfac.mu-sofia.bg.

1. Do you agree to participate in this survey, and to answer the questions anonymously?
    - Yes
    - No

2. Your sex:
    - Male
    - Female

3. What is your age?
   ....

4. Marital status:
    - Married
    - Unmarried
    - Divorced
    - Widow / widower

5. What is the length of service as a pharmacist (in years)?
   ....

6. What is your income working as a pharmacist?
    - Less than 600 Euro
    - 600-1000 Euro
    - More than 1000 Euro

7. Do you have a specialty in the pharmaceutical area?
    - Yes
    - No

8. If yes, please choose your specialty:
    - General Pharmacy
    - Clinical Pharmacy
    - Pharmaceutical Industry
    - Pharmaceutical Laboratory
    - Other

9. Have you used food supplements with vitamin C during the pandemic of COVID19?
    - Yes
    - No

10. Have you recommended food supplements with vitamin C to your family/friends/patients during the pandemic of COVID19?
    - Yes
    - No

11. Have you used food supplements with vitamin D during the pandemic of COVID19?
    - Yes
    - No

12. Have you recommended food supplements with vitamin D to your family/friends/patients during the pandemic of COVID19?
    - Yes
    - No

13. Would you get vaccinated against COVID19?
    - Yes
    - No

QUALITY OF LIFE QUESTIONNAIRE (15D©)

Please read through all the alternative responses to each question before placing a cross (x) against the alternative which best describes **your present health status**. Continue through all 15 questions in this manner, giving only **one** answer to each.

QUESTION 1. MOBILITY

1 ( ) I am able to walk normally (without difficulty) indoors, outdoors and on stairs.
2 ( ) I am able to walk without difficulty indoors, but outdoors and/or on stairs I have slight difficulties.
3 ( ) I am able to walk without help indoors (with or without an appliance), but outdoors and/or on stairs only with considerable difficulty or with help from others.
4 ( ) I am able to walk indoors only with help from others.
5 ( ) I am completely bed-ridden and unable to move about.

QUESTION 2. VISION

1 ( ) I see normally, i.e. I can read newspapers and TV text without difficulty (with or without glasses).
2 ( ) I can read papers and/or TV text with slight difficulty (with or without glasses).
3 ( ) I can read papers and/or TV text with considerable difficulty (with or without glasses).
4 ( ) I cannot read papers or TV text either with glasses or without, but I can see enough to walk about without guidance.
5 ( ) I cannot see enough to walk about without a guide, i.e. I am almost or completely blind.

QUESTION 3. HEARING

1 ( ) I can hear normally, i.e. normal speech (with or without a hearing aid).
2 ( ) I hear normal speech with a little difficulty.
3 ( ) I hear normal speech with considerable difficulty; in conversation I need voices to be louder than normal.
4 ( ) I hear even loud voices poorly; I am almost deaf.
5 ( ) I am completely deaf.

QUESTION 4. BREATHING

1 ( ) I am able to breathe normally, i.e. with no shortness of breath or other breathing difficulty.
2 ( ) I have shortness of breath during heavy work or sports, or when walking briskly on flat ground or slightly uphill.
3 ( ) I have shortness of breath when walking on flat ground at the same speed as others my age.
4 ( ) I get shortness of breath even after light activity, e.g. washing or dressing myself.
5 ( ) I have breathing difficulties almost all the time, even when resting.

QUESTION 5. SLEEPING

1 ( ) I am able to sleep normally, i.e. I have no problems with sleeping.
2 ( ) I have slight problems with sleeping, e.g. difficulty in falling asleep, or sometimes waking at night.
3 ( ) I have moderate problems with sleeping, e.g. disturbed sleep, or feeling I have not slept enough.
4 ( ) I have great problems with sleeping, e.g. having to use sleeping pills often or routinely, or usually waking at night and/or too early in the morning.
5 ( ) I suffer severe sleeplessness, e.g. sleep is almost impossible even with full use of sleeping pills, or staying awake most of the night.

QUESTION 6. EATING

1 ( ) I am able to eat normally, i.e. with no help from others.
2 ( ) I am able to eat by myself with minor difficulty (e.g. slowly, clumsily, shakily, or with special appliances).
3 ( ) I need some help from another person in eating.
4 ( ) I am unable to eat by myself at all, so I must be fed by another person.
5 ( ) I am unable to eat at all, so I am fed either by tube or intravenously.

QUESTION 7. SPEECH

1 ( ) I am able to speak normally, i.e. clearly, audibly and fluently.
2 ( ) I have slight speech difficulties, e.g. occasional fumbling for words, mumbling, or changes of pitch.
3 ( ) I can make myself understood, but my speech is e.g. disjointed, faltering, stuttering or stammering.
4 ( ) Most people have great difficulty understanding my speech.
5 ( ) I can only make myself understood by gestures.

QUESTION 8. EXCRETION

1 ( ) My bladder and bowel work normally and without problems.
2 ( ) I have slight problems with my bladder and/or bowel function, e.g. difficulties with urination, or loose or hard bowels.
3 ( ) I have marked problems with my bladder and/or bowel function, e.g. occasional 'accidents', or severe constipation or diarrhea.
4 ( ) I have serious problems with my bladder and/or bowel function, e.g. routine 'accidents', or need of catheterization or enemas.
5 ( ) I have no control over my bladder and/or bowel function.

QUESTION 9. USUAL ACTIVITIES

1 ( ) I am able to perform my usual activities (e.g. employment, studying, housework, free-time activities) without difficulty.
2 ( ) I am able to perform my usual activities slightly less effectively or with minor difficulty.
3 ( ) I am able to perform my usual activities much less effectively, with considerable difficulty, or not completely.
4 ( ) I can only manage a small proportion of my previously usual activities.
5 ( ) I am unable to manage any of my previously usual activities.

QUESTION 10. MENTAL FUNCTION
1 ( ) I am able to think clearly and logically, and my memory functions well
2 ( ) I have slight difficulties in thinking clearly and logically, or my memory sometimes fails me.
3 ( ) I have marked difficulties in thinking clearly and logically, or my memory is somewhat impaired.
4 ( ) I have great difficulties in thinking clearly and logically, or my memory is seriously impaired.
5 ( ) I am permanently confused and disoriented in place and time.

QUESTION 11. DISCOMFORT AND SYMPTOMS
1 ( ) I have no physical discomfort or symptoms, e.g. pain, ache, nausea, itching etc.
2 ( ) I have mild physical discomfort or symptoms, e.g. pain, ache, nausea, itching etc.
3 ( ) I have marked physical discomfort or symptoms, e.g. pain, ache, nausea, itching etc.
4 ( ) I have severe physical discomfort or symptoms, e.g. pain, ache, nausea, itching etc.
5 ( ) I have unbearable physical discomfort or symptoms, e.g. pain, ache, nausea, itching etc.

QUESTION 12. DEPRESSION
1 ( ) I do not feel at all sad, melancholic or depressed.
2 ( ) I feel slightly sad, melancholic or depressed.
3 ( ) I feel moderately sad, melancholic or depressed.
4 ( ) I feel very sad, melancholic or depressed.
5 ( ) I feel extremely sad, melancholic or depressed.

QUESTION 13. DISTRESS
1 ( ) I do not feel at all anxious, stressed or nervous.
2 ( ) I feel slightly anxious, stressed or nervous.
3 ( ) I feel moderately anxious, stressed or nervous.
4 ( ) I feel very anxious, stressed or nervous.
5 ( ) I feel extremely anxious, stressed or nervous.

QUESTION 14. VITALITY
1 ( ) I feel healthy and energetic.
2 ( ) I feel slightly weary, tired or feeble.
3 ( ) I feel moderately weary, tired or feeble.
4 ( ) I feel very weary, tired or feeble, almost exhausted.
5 ( ) I feel extremely weary, tired or feeble, totally exhausted.

QUESTION 15. SEXUAL ACTIVITY
1 ( ) My state of health has no adverse effect on my sexual activity.
2 ( ) My state of health has a slight effect on my sexual activity.
3 ( ) My state of health has a considerable effect on my sexual activity.
4 ( ) My state of health makes sexual activity almost impossible.
5 ( ) My state of health makes sexual activity impossible.
